# Audit on Preoperative Fasting of Elective Surgical Patients in an African Academic Medical Center

**DOI:** 10.1007/s00268-014-2582-3

**Published:** 2014-04-21

**Authors:** Endale Gebreegziabher Gebremedhn, Vidhya Bates Nagaratnam

**Affiliations:** Department of Anaesthesia, School of Medicine, College of Medicine and Health Sciences, University of Gondar, P.O. Box 196, Gonder, Ethiopia

## Abstract

**Background:**

Preoperative fasting is a requisite before anesthesia. The main reason for preoperative fasting is to reduce gastric volume and acidity and thus decrease the risk of pulmonary aspiration. However, preoperative fasting is usually prolonged beyond the recommended time for various reasons. Despite the many adverse effects of prolonged fasting, patients sometimes fasted for a prolonged time when surgery was delayed for different reasons at the University of Gondar Hospital. The aim of this study was to assess the duration of preoperative fasting for elective surgery.

**Methods:**

A cross-sectional study was conducted from March 10 to April 10, 2013. Patients were interviewed 24 h after surgery. All 43 patients who were under anesthesia while operated on during the study period were included.

**Result:**

Of the 43 patients included in the study, 35 were adults and 8 were children. The minimum, maximum, and mean fasting hours for food were 5, 96, and 19.60, respectively, and more than 50 % of the patients fasted from food twice as long as recommended. The minimum, maximum, and mean fasting hours for fluid were 5, 19, and 12.72, respectively. More than 95 % of the patients fasted from fluid longer than recommended.

**Conclusion:**

Most patients fasted from both food (92 %) and fluid (95 %) longer than the fasting time recommended by the AAGBI, ASA, RCOA, and RCN fasting guidelines. Anesthetists, surgeons, and nurses need to revise operation lists every day in the operating theatres and resuscitate the patients when surgery is delayed for various reasons. A preoperative fasting guideline should be developed and implemented in the University of Gondar Hospital.

## Introduction

Anesthesia-related pulmonary aspiration that may lead to respiratory failure has been mentioned in both elective and emergency surgery patients. Fluid and food intake by healthy and elective surgery patients may increase aspiration risk by increasing residual gastric volume, decreasing pH, and decreasing esophageal sphincter tone. General anesthesia also reduces reflexes that prevent regurgitation and aspiration of gastric contents. Therefore, fasting is ordered before surgery. Conventionally, preoperative fasting is advised to keep patients nil per os (NPO) from food and drink from midnight before the day of surgery to minimize the risk of pulmonary aspiration [1–3].

Much evidence suggests that a normal and healthy stomach empties clear fluids rapidly so it is safe to drink clear fluids up to 2 h before the induction of anesthesia, and solid food is permitted up to 6 h before surgery. Therefore, the order NPO starting at midnight for elective surgery can be adjusted to no intake of solid food and milk products 6 h before surgery and fast from clear fluid 2 h before surgery [4–10].

There are different challenges for the implementation of fasting guidelines in Ethiopia. The main ones are lack of a national and/or a local fasting protocol, lack of knowledge among health professionals about the advantage of following internationally accepted fasting guidelines and the adverse effect of prolonged fasting, lack of updating training, absence of an enforcing committee, and lack of auditing in the hospitals.

It is clear that developing a national and/or local fasting protocol, training about the advantage of using globally accepted fasting guidelines and the adverse effects of prolonged fasting, launching an enforcing committee, and auditing the adherence of health professionals to the fasting guidelines after implementation will enable the surgical team to think ahead and rearrange the operation schedule lists concerning preoperative food and/or fluid management of the patients. These actions will also help to avoid or, at least, minimize the adverse effects of prolonged fasting, improve the patient’s postoperative outcome, and improve the patient’s experience with anesthesia and surgery.

The University of Gondar referral and teaching hospital provides health services for more than five million people and 5,360 patients were operated on while under anesthesia in 2005, according to the annual report of the Department of Anaesthesia.

There was a big gap between the time the patients started NPO as ordered and the time the operation was performed. There was also little concern among the surgical team for prolonged starvation of patients before surgery despite its adverse effects like hunger, thirst, dehydration, and hypoglycemia. Hence, the aim of this audit was to assess preoperative fasting duration by patients who were scheduled for elective surgery at the University of Gondar teaching hospital (Table 1).

**Table 1:** Guidelines used as reference for this clinical audit (AAGBI, ASA, RCOA, and RCN)

Ingested material	Minimum fast (h)
Clear fluids	2
Breast milk	4
Infant formula	6
Nonhuman milk	6
Light meal	6

## Materials and methods

### Study design and period

A quantitative cross-sectional study design was used and the study was conducted from March 10 to April 10, 2013.

### Audit population

All elective (adult and children) patients who operated under anaesthesia during the data collection period were included.

### Audit sample

Fourty three elective patients who operated under anaesthesia and met the inclusion criteria during the data collection period were included.

### Data collection method

A checklist was developed that addressed patient identity, type of operation (minor vs. major, morning vs. afternoon), time the operation started, fasting status [duration of fasting from food and fluid, preoperative maintenance fluid status, advice received, hunger and thirst score (0–4)], change to the order of the operation schedule lists and patient awareness about existing fasting guidelines. The checklist was pilot tested in other hospitals and changes were made before data collection. Patients were visited the night before and the morning of the day of surgery. They were interviewed about the time of NPO after 24 h after surgery. For patients younger than 15 years old, the parent or the guardian was interviewed. The actual time that surgery started was also obtained from the patients’ charts and the anesthetic record sheets after the operation. Permission for this clinical audit was obtained from the institutional ethical review board, College of Medicine and Health Sciences, University of Gondar.

## Results

### Sociodemographic characteristics of respondents

A total of 52 elective surgery patients were operated on during the study period. Of these, six minor surgery and three major surgery patients were discharged before 24 h after surgery. Thus, 43 (82.7 %) of the 52 elective surgery patients were included in our audit. Males accounted for 55.8 % of the patients and 58.1 % of the patients were between 30 and 49 years old. Twenty-one (48.8 %) of the 43 patients were not able to write and read. The majority (95.3 %) of the operations were major (Tables 2, 3).

**Table 2:** Age of elective surgery patients who were operated on under anesthesia at the University of Gondar Hospital, March 10–April 10, 2013 (*N* = 43)

Age (year)	Frequency	Percentage
<1	1	2.3
1–5	0	0
6–18	7	16.3
19–29	7	16.3
30–49	25	58.1
50–65	0	0
>65	3	7.0
Total	43	100.0

**Table 3:** Educational status of elective surgery patients who were operated on under anesthesia at the University of Gondar Hospital, March 10–April 10, 2013 (*N* = 43)

Educational status	Frequency	Percentage
Not applicable (children)	2	4.7
Illiterate	21	48.8
Primary education	16	37.2
Secondary education	4	9.3

### Factors related to preoperative fasting

All patients were given instructions about preoperative fasting but no patient was given information about the available preoperative fasting guidelines. There was no change in the order of the operation schedule lists during the data collection period.

The minimum, maximum, and mean fasting hours for food were 5, 96, and 19.60, respectively. More than 50 % of the patients fasted from food two times longer than the recommended preoperative fasting times of the Association of Anaesthetists of Great Britain and Ireland (AAGBI), American Society of Anesthesiologists (ASA), Royal College of Anaesthetists (RCOA), and Royal College of Nursing (RCN) fasting guidelines (Table 4). The minimum, maximum, and mean fasting hours from fluid were 5, 19, and 12.72, respectively. More than 95 % of the patients fasted from fluid longer than the recommended preoperative fasting times of the AAGBI, ASA, RCOA and RCN fasting guidelines (Table 5).

**Table 4:** Fasting time for food for elective surgery patients who were operated on under anesthesia at the University of Gondar Hospital, March 10–April 10, 2013 (*N* = 43)

Duration of fasting from food (h)	Frequency	Percentage
2	0	0
3–4	1	2.3
5–6	2	4.7
7–8	0	0
9–12	15	34.9
>12	25	58.1

**Table 5:** Fasting time for fluid for elective surgery patients who were operated on under anesthesia at the University of Gondar Hospital, March 10–April 10, 2013 (*N* = 43)

Duration of fasting from fluid (h)	Frequency	Percentage
2	0	0
3–4	0	0
5–6	1	2.3
7–8	1	2.3
9–12	20	46.5
>12	21	48.8

A larger number (49 %) of patients experienced slight to severe thirst because of prolonged fasting from fluid. There was also a relationship between long fasting from fluid and degree of thirst: 47 % of the patients experienced slight to severe thirst as the duration of fasting lasted beyond the recommended time (Tables 6, 7).

**Table 6:** Degree of thirst for elective surgery patients who were operated on under anesthesia at the University of Gondar hospital, March 10–April 10, 2013 (*N* = 43)

Degree of thirst	Frequency	Percentage
No	22	51.2
Slight thirst	1	2.3
Mild thirst	5	11.6
Moderate thirst	10	23.3
Severe thirst	5	11.6

**Table 7:** Relationship between the duration of fasting from fluid and degree of thirst for elective surgery patients who were operated on under anesthesia at the University of Gondar Hospital, March 10–April 10, 2013 (*N* = 43)

Duration of fasting from fluid (h)	Degree of thirst				
	No	Slight	Mild	Moderate	Severe
2	0	0	0	0	0
3–4	0	0	0	0	0
5–6	0	0	0	1	0
7–8	0	0	0	1	0
9–12	8	1	3	6	2
>12	14	0	2	2	3

Many patients (37.2 %) experienced mild to severe hunger due to prolonged fasting from food. The degree of hunger was markedly related to the duration of fasting from food: 33 % of the patients experienced mild to severe hunger as the duration of fasting lasted beyond the recommended time (Tables 8, 9)..

**Table 8:** Degree of hunger for elective surgery patients who were operated on under anesthesia at the University of Gondar Hospital, March 10–April 10, 2013 (*N* = 43)

Degree of hunger	Frequency	Percentage
No	27	62.8
Slight hunger	0	0
Mild hunger	7	16.3
Moderate hunger	5	11.6
Severe hunger	4	9.3

**Table 9:** Relationship between the duration of fasting from food and degree of hunger for elective surgery patients who were operated on under anesthesia at the University of Gondar Hospital, March 10–April 10, 2013 (*N* = 43)

Duration of fasting from food (h)	Degree of hunger				
	No	Slight	Mild	Moderate	Severe
2	0	0	0	0	0
3–4	1	0	0	0	0
5–6	0	0	1	0	1
7–8	0	0	0	0	0
9–12	12	0	1	1	1
>12	14	0	5	4	2

Duty nurses and interns put 33 (4 of which were children) patients on fluids starting the night before surgery. Surprisingly, all of these patients showed up with the first IV fluid bag almost full (without getting adequate intravenous fluid overnight) the morning of surgery. There was no difference in the severity of hunger and thirst between the group of patients who were put on maintenance fluid and those who were not on maintenance fluid. In this clinical audit, we found that the severity of hunger and thirst for children with respect to the duration of preoperative fasting was not different than that of the adult patients.

## Discussion

This clinical audit showed that most of the patients fasted from food and fluid longer than the time recommended by most of the international guidelines. This might have many adverse consequences such as hunger, thirst, headache, dehydration, hypoglycemia, delayed awakening after anesthesia, and poor patient outcome after surgery. The main reasons for the prolonged fasting in our hospital might be the tradition of ordering patients to be NPO starting at midnight and the lack of a local fasting protocol. In addition, there was no trend of revising the operation lists and ordering patients to take food and/or fluid whenever surgery was delayed. Although it was imperative to audit children and adult patients separately, we combined the data from the children and the adults to find the magnitude of the problem. These data showed that most of the time patients were fasting for prolonged period, and there was no concern about the duration of fasting and the sequence of operation schedule lists for children in our hospital by the surgical team whenever surgery was delayed. However, the severity of hunger and thirst of the children was not different from that of the adult patients; this could be due to small audit sample and the small number of pediatric patients included in our audit. The severe hunger and thirst resulted in a bad preoperative experience for the children in contrast to other studies where children who were allowed to take fluid and food within the recommended time were comfortable [1]. There also was no change in the order of operations lists during the data collection period and no patient was given information about the available fasting guidelines.

The minimum, maximum, and mean fasting hours for food were 5, 96, and 19.60, respectively. More than 50 % of the patients fasted from food two times longer than the recommended preoperative fasting times of the AAGBI, ASA, RCOA, and RCN fasting guidelines. Many patients (37.2 %) experienced mild to severe hunger due to prolonged fasting from food. The degree of hunger was markedly related to the duration of fasting from food (33 % of patients experienced mild to severe hunger as the duration of fasting went beyond the recommended time). This finding was similar to that of another study where preoperative fasting was prolonged compared with other guidelines [11, 12]. This huge deviation from the known fasting guidelines is an alarming problem for our hospital clinical practice and is caused by the traditional preoperative instructions given to the patients to be NPO starting at midnight irrespective of age. This is an outdated order as evidenced by many previous studies.

Although preoperative clear fluid intake 2 h before surgery is recommended by different guidelines and found to be safe for the patient, fluid fasting is prolonged due to fear by anesthetists of pulmonary aspiration [13, 14]. The minimum, maximum, and mean fasting hours from fluid in our study were 5, 19, and 12.72, respectively. More than 95 % of the patients fasted from fluid longer than the recommended preoperative fasting times of the AAGBI, ASA, RCOA and RCN guidelines. A larger number (49 %) of patients experienced slight to severe thirst because of prolonged fasting from fluid. There was a relationship between long duration of fasting from fluid and degree of severity of thirst (47 % of the patients experienced slight to severe thirst as the duration of fasting time went beyond the recommended time).This finding was in line with findings of other studies [11, 12] where preoperative fasting was longer than the recommended time.

In this audit, even though duty nurses and interns put most patients (33/43) on fluids starting the night before the day of surgery, surprisingly all of these patients showed up with the first IV fluid bag almost full (and did not get adequate IV fluid overnight) the morning of surgery. We wanted to go to the wards at different time intervals within 24 h of surgery to check how much fluid each patient was given but this was not possible once we recognized this gap after collecting half of the data. A limitation of this audit was that although most patients were on maintenance fluid before surgery, we did not quantify the amount of fluid each patient was given during the fasting window.

## Conclusion

The majority of patients fasted from both food and fluid longer than the fasting time recommended by the AAGBI, ASA, RCOA and RCN guidelines. There was no trend by anesthetists, surgeons, and operating theatre nurses of revising the schedule lists and ordering patients to take a light meal or fluid whenever the surgery delayed. Anesthetists, surgeons, and nurses need to revise and discuss the scheduled lists every day in the operating theatres and resuscitate the patients accordingly.

Preoperative fasting guidelines for both food and fluid should be developed and implemented in the University of Gondar Hospital operation theatres and wards. Anesthetists, surgeons, and nurses should be made aware of the current problem and the adverse effect of prolonged fasting. Although our audit sample was small, it seems sensible to recommend auditing preoperative fasting of elective surgery patients in other hospitals in Ethiopia since our fasting times were, by far, more prolonged than the recommended times.
